# New Mode of Administering Cod-Liver Oil

**Published:** 1851-08

**Authors:** 


*New mode of administering Cod-Liver Oil.*—M. Loze, a naval surgeon, stated that he had attributed the failures of this remedy in phthisis to its non-absorption; and that, with the view of effecting this, he had combined the oil with a mucilage containing pancreatic juice, which thus forms an artificial chyle. In this form the oil is readily and completely absorbed, and its effect more directly obtained in phthisis.—*London Medical Gazette.*

